# Trends of nutrients and metals in precipitation in northern Germany: the role of emissions and meteorology

**DOI:** 10.1007/s10661-021-09094-y

**Published:** 2021-05-05

**Authors:** Malte Lorenz, Matthias Brunke

**Affiliations:** 1 Landesamt für Landwirtschaft, Umwelt und ländliche Räume des Landes Schleswig-Holstein, Abteilung Gewässer, Dezernat Fließgewässerökologie, Hamburger Chaussee 25, Flintbek, 24220 Germany; 2 Landesamt für Umwelt Rheinland-Pfalz, Abteilung Gewässerschutz, Kaiser-Friedrich-Straße 7, Mainz, 55116 Germany

**Keywords:** Atmospheric deposition, Trend analysis, Multivariate statistics, Time series decomposition, Heavy metals, Nutrients

## Abstract

We analyzed the precipitation chemistry for a maritime region in northern Germany (Schleswig–Holstein) from 1997 to 2017 in order to reveal temporal and spatial patterns and to evaluate the role of meteorological factors relative to emission reductions in Germany and Europe. Therefore, we applied several statistical methods such as time series decomposition, principal component, and redundancy analysis. We extracted two main groups: (i) a marine group (Cl, Na, Mg) that was related to natural processes like sea spray input and (ii) an anthropogenic group (Pb, Cd, As, Zn, and nitrogen species) with a terrestrial subgroup (Fe, Al, Mn), which were both related to emissions. These groups were valid for the spatial, seasonal, and annual trend data. Other elements, like Ca, K, total P, and sulfate, were influenced by natural and anthropogenic processes. The seasonal variation of ammonium deposition was caused primarily by ammonia emissions and ancillary by precipitation. Most heavy metals as well as sulfate, nitrate, and ammonium showed decreasing trends in concentrations and deposition fluxes. Only Hg did not show any trend. The decreasing depositions of sulfate and total nitrogen were correlated to emission reductions in Germany. The deposition of most heavy metals was influenced by emission reductions on European scale and meteorological factors such as wind speed and humidity. Hg did not show any correlation with the emission time series in Europe. Instead, it was correlated to the NAO index and wind, implying that global emissions and transport pathways determine the temporal development of Hg depositions. Overall, the study reveals that emission reductions positively influence regional depositions for most investigated substances. The regional spatial patterns of depositions were also influenced by local meteorological factors.

## Introduction

The alteration of the atmospheric deposition, due to human emissions of particulate matter, heavy metals, nutrients, and other anthropogenic pollutants, is a global problem (Monks et al., 2009). The excessive deposition of sulfate, ammonium, and nitrate leads to an acidification and eutrophication of ecosystems, which can cause changes in biodiversity (Pascaud et al., 2016). In addition, the deposition and enrichment of heavy metals, like Pb, Cd, and Hg, in ecosystems are a major environmental concern due to their persistence, ability to bio-accumulate, and their toxicological effects (Tørseth et al., 2012).

Anthropogenic sulfur and nitrogen emissions mainly originate from energy production (SO_x_ and NO_x_), road transportation (NO_x_), and agriculture (NH_3_) (Zhang et al., 2018). Some of the most important emission sources for heavy metals are the metal industry (Al, As, Cr, Cu, Fe, Zn), other manufacturing industries and construction (As, Cd, Cr, Hg, Ni, Pb), electricity and heat production (Hg, Ni), road transportation (Cu from brake wear, Pb from petrol, and Zn from tires), and phosphate fertilizers in agricultural areas (Cd) (Huang et al., 2009; Schröder et al., 2016).

NO_x_ and SO_2_ emissions are decreasing in Europe and in the USA, but the rate of change is smaller in the USA, particularly for NO_x_ (Monks et al., 2009; Zhang et al., 2018). On a global scale, these emission reductions are compensated by increasing emissions in East Asia (Monks et al., 2009). In Europe, emissions of SO_x_, NO_x_, and most heavy metals have been reduced, since 1990, by national and international conventions and mitigation measures, like the UNEC Convention on Long-Range Transboundary Air Pollution (LRTAP) (UNECE, 1979).

Despite different analysis periods in the time 1990 to 2010, several authors found declining sulfur and nitrogen concentrations and deposition fluxes in Europe since 1990, in line with emission reduction policies (Aas et al., 2019; Tørseth et al., 2012; van der Swaluw et al., 2011; Vet et al., 2014; Waldner et al., 2014). Although on a national or regional scale, deviations from the general European trend occur (Hůnová et al., 2014; Pascaud et al., 2016; Winfried Schröder et al., 2014). For example, Pascaud et al. (2016) found decreasing trends in sulfate and hydrogen concentration at a large number of rural sites in France, but the trends in nitrogen compounds were not linked to emission inventory changes.

In addition, in the same period, long-term trends in heavy metal concentrations (Pb and Cd) and deposition fluxes decreased since 1990 in Europe (Pacyna et al., 2009; Tørseth et al., 2012). Schröder et al. (2016) compared heavy metal long-term trends in mosses with EMEP (European Monitoring and Evaluation Programme) domain depositions and found decreasing trends for Pb, Cd, Cr, Zn, Ni, Fe, As, Hg, and Cu following emission reductions. Despite falling Hg emissions in Europe, no or only minor changes have been observed in Hg concentrations and deposition fluxes, depending on the investigated time periods (Torseth, 2012; Schröder et al., 2016; Pacyna et al., 2009). For all waters in Germany, Hg concentrations in river biota exceed the environmental quality standard defined by the EU water framework directive (BMUB/UBA, 2016). Direct Hg releases to surface waters have been greatly reduced over the last decades, and today, inputs are dominated by diffuse sources (e.g., atmospheric deposition, soil erosion, and the remobilization) (Wiederhold et al., 2020). The long live time of Hg in the atmosphere resulted in an increased focus on global Hg emission sources (HTAP, 2010; UNEP, 2013).

The magnitude of atmospheric deposition can be influenced by many factors such as the temporal development and spatial distribution of emission sources and meteorological factors (e.g., wind speed, temperature, or humidity) (Amodio et al., 2014; Mijić et al., 2010; Suvarapu & Baek, 2017). Heavy metals, such as Pb, Cd, and Hg, as well as sulfur and nitrogen aerosols, can be transported over long distances by atmospheric flow, before they are deposited far away from the emission sources (Pacyna et al., 1989). Therefore, it is sometimes equivocal, if observed trends in atmospheric deposition can be solely attributed to changes in emissions, because temporal changes in meteorological factor can further support or contradict changes.

The monitoring of precipitation chemistry often results in complex data sets that comprise a large number of physical–chemical parameters. However, simultaneous evaluation and interpretation of multiple parameters of such data sets was found to be difficult (Le et al., 2017). Consequently, there is a demand to apply approaches that provide deeper insights into complex environmental and anthropogenic dependencies.

In this study, a comprehensive and complex data set of deposition measurements of 24 chemical parameters, including major ions, nitrogen, and phosphorous species as well as heavy metals, has been analyzed for a maritime region in northern Germany. To explore the relationships between the parameters and furthermore the relationships of the parameters to another data set of environmental variables, canonical ordination methods were applied (Borcard et al., 2011). Although multivariate constrained ordination methods, like redundancy analysis (RDA), and variation partitioning are growing in popularity in ecological analysis and modelling (Dalu et al., 2017; Rico et al., 2016), they were rarely applied in other disciplines.

The first objective of the study was to explore the spatial and seasonal variation as well as trends of nutrients and heavy metal concentrations in precipitation and their deposition loads. The second objective of the study was to gain deeper insight in the relationships between data sets of deposition measurements, emission inventories, and meteorological factors, which were addressed by advanced multivariate ordination methods. Therefore, time series decomposition algorithms were used together with multivariate analysis methods to gain insight in: (1) the spatial and seasonal variation, (2) the temporal patterns (long-term trends), as well as (3) the relative influence of the temporal development of emission inventories and meteorological factors on the temporal development of selected parameters.

## Methods

### Sampling sites and chemical analysis

**Fig. 1:**
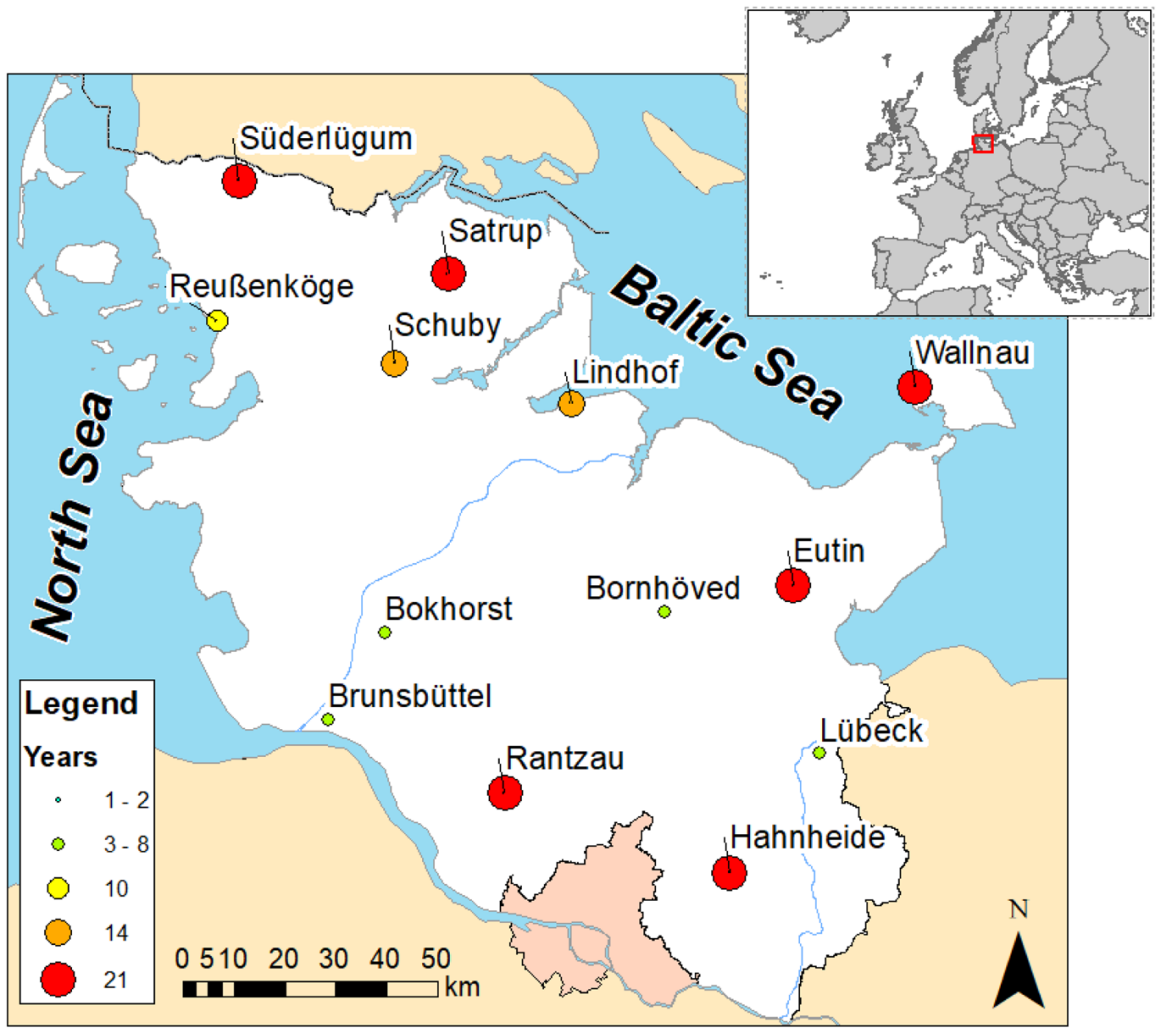
Location and names of the sampling sites. The color and size of the symbols indicates the length of the available time series for each site

The 12 sampling sites of this study are located in the federal state Schleswig–Holstein in northern Germany (Fig. 1). Schleswig–Holstein is the most northern part of Germany, enclosed by the North Sea in the West and the Baltic Sea in the East. Geographically, this region is part of the North German Lowlands and is characterized by an oceanic climate. The annual mean temperature is 8.3 °C and the wind direction is dominated by prevailing westerlies (Beyn et al., 2014).

All sampling sites were equipped with automatic precipitation collectors (OTT Pluvio 2), bulk samplers, and automatic wet-only samplers. Bi-weekly composite samples were collected from the wet-only samplers to analyze nutrients (NH_4_^+^, NO_2_^−^, NO_3_^−^, total nitrogen (N), and total phosphorous (P)) as well as major ions (Cl^−^, Na^+^, K^+^, Ca^2+^, Mg^2+^, SO_4_^2−^) and dissolved organic carbon (DOC). Four-week composite samples were collected from the bulk samplers to analyze heavy metals (Fe, Mn, As, Cd, Cr, Pb, Cu, Ni, Zn, and Hg) and Al. The samples were analyzed in the state laboratory Schleswig–Holstein (Table 1). The quality assurance of the chemical analyses was continuously realized by the annual participation at the laboratory ring tests at the Norwegian Institute for Air Research (NILU). Secondly, the laboratory had participated at ring tests of the European Monitoring and Evaluation Programme (EMEP) up to now for 38 times.

**Table 1:** List of investigated substances and applied analytical methods

Parameter	Method
pH	Potentiometric
NH_4_^+^, NO_2_^−^, NO_3_^−^, total N, and total P	Continuous flow analyzer
SO_4_^2−^, Cl^−^	Flow injection analyzer
Na^+^, K^+^, Ca^2+^, Mg^2+^, Al, Fe, Mn, As, Cd, Cr, Pb, Cu, Ni, Zn	ICP-MS/MS, ICP-OES
Hg	AFS
DOC	TOC analyzer

The time series of these sites were used for the seasonal and the trend analysis. At five sites, time series of 21 years (1997 to 2017) were available (Fig. 1). For the spatial analysis, the available data of all sites in the time period of 2012 to 2017 were used.

### Data processing 

Prior to data aggregation, three criteria were used to identify inappropriate data for analysis: (1) samples contaminated with dust or bird droppings, (2) data higher than mean plus three times the standard deviation, and (3) precipitation volume < 1 mm (Huang et al., 2008). Approximately 2% of the data were excluded from analyses according to the above criteria.

The time series were aggregated to monthly time series calculating volume-weighted mean concentrations (*C*_VW_) according to Eq. 1:

1 $${C}_{\mathrm{VW}}= \frac{\sum_{i=1}^{n}\left({C}_{\mathrm{i}} {P}_{\mathrm{i}}\right)}{\sum_{i=1}^{n}{P}_{\mathrm{i}}}$$

where *n* is the number of samples, *C*_i_ is the concentration, and *P*_i_ is the precipitation measured by the precipitation collector. The associated deposition fluxes (*D*) were calculated following Eq. 2:

2 $$D= \sum_{i=1}^{n}\left({C}_{\mathrm{i}} {P}_{\mathrm{i}}\right)$$

The sea salt (*ss*) and non-sea salt ratios (*nss*) of the ions were estimated by Eqs. 3 and 4 (Honoki et al., 2007; Keene et al., 1986) based on sodium and the ionic concentration ratio of seawater expressed in eq/eq

3 $$ss{I}_{\mathrm{i}}={Na}^{+} {\left({I}_{\mathrm{i}}/{Na}^{+}\right)}_{seawater}$$

where *I*_i_ is the concentration of one ion, e.g., SO_4_^2−^

4 $$nss{I}_{\mathrm{i}}= {I}_{\mathrm{i}}- {Na}^{+} {\left({I}_{\mathrm{i}}/{Na}^{+}\right)}_{seawater}$$

Additionally to the inter laboratory comparisons and quality checks carried out by the laboratory, checks on ion balance, comparison between measured and calculated conductivity, and Na/Cl ratio were performed (Mosello et al., 2005) (Table 2).

**Table 2:** Summary of the percentage difference (PD) of the ion balance, Na + /Cl^−^ ratio, and percentage difference (CD) of calculated (CE) vs. measured conductivity (CM). Units: ion concentrations µeq l^−1^ and conductivity µS cm^−1^

Site	Sum Cat	Sum An	PD	Na^+^/Cl^−^	CE	CM	CD
Bokhorst	0.182	0.163	10.9	0.91	28.3	25.5	10.9
Bornhöved	0.158	0.141	10.9	0.90	25.0	25.4	−1.6
Brunsbüttel	0.214	0.200	6.7	0.81	35.5	33.2	7.0
Hahnheide	0.124	0.119	3.9	0.85	23.6	25.5	−7.3
Hennstedt	0.297	0.287	3.5	0.82	37.2	34.4	8.0
Lindhof	0.163	0.159	2.5	0.80	26.8	29.9	−10.5
Lübeck	0.120	0.116	3.7	0.86	20.5	20.6	−0.6
Rantzau	0.144	0.134	7.1	0.84	23.3	26.9	−13.4
Reußenköge	0.260	0.247	5.3	0.83	40.5	38.4	5.6
Satrup	0.151	0.149	1.1	0.80	25.3	28.7	−11.8
Schuby	0.205	0.195	5.1	0.85	32.3	34.5	−6.4
Süderlügum	0.192	0.191	0.2	0.85	31.5	32	−1.5
Wallnau	0.173	0.167	3.4	0.81	28.4	30.7	−7.4
Sites mean			4.9	0.84	29.1		−1.9

The check of the ion balance is based on the test of electronegativity of water samples. The ion balance is evaluated by the percentage difference (PD) of the sum of cations and sum of anions expressed in µeq l^−1^. The PD should be in the range + / − 10%.

5 $$PD= 100* \frac{\sum Cat-\sum An}{0.5*\left(\sum Cat+\sum An\right)}$$

The PD shows a range of 0.2 to 10.9 and the mean PD over all stations is 4.9 and is considered as acceptable.

The percentage difference (CD) between measured (CM) and calculated (CE) conductivity is given by:

6 $$CD= 100* \frac{CE-CM}{CM} with \ CE= {\lambda }_{\mathrm{i}}{C}_{\mathrm{i}}$$

where *CE* [µS cm^−1^] is the sum of the concentrations [µeq l^−1^] and $\lambda$ [kS cm^2^ eq^−1^] is the equivalent conductance at infinite dilution of the ion i. Conversion factors are listed in Mosello et al. (2005). The CD should be in the range + / − 10%. The CD shows a range of − 13.4 to 10.9 and the mean CD over all stations is 1.9 and is considered as acceptable.

The ratio between Na^+^/Cl^−^ should be expected not far from the marine value 0.89 in a range of 0.5 to 1.5 (Keene et al., 1986; Mosello et al., 2005). The CD should be in the range + / − 10%. The ratio between Na^+^/Cl^−^ shows a range of 0.8 to 0.91 with a mean ratio over all stations 0.84 and is considered as acceptable.

### Statistical methods

Since strong seasonal variation can mask trends in parameters, the seasonal trend decomposition procedure based on non-parametric local regression (STL) was applied to extract a seasonal and trend component from the original time series (Cleveland et al., 1990). Based on the STL method, every data point in a time series is the sum of three independently interpretable components: (1) the seasonal component (high frequency), (2) the trend component (long-term change or low frequency), and (3) residual or random component. The STL method extracts the seasonal und trend component by an iterative local regression (LOESS) algorithm (Zobrist et al., 2018). A window width of 3 years for the seasonal and 6 years for the trend component was chosen for the smoothing parameters. A distinct longer smoothing period, such as 10 years, would mask meaningful information (Zobrist et al., 2018). The STL decomposition was performed for the five sites with 21 years of data (Fig. 1). The averaged time series over all sites is denoted as station mean. The extracted seasonal and trend component time series allow a separate evaluation, respectively. A data matrix for the spatial analysis was created by averaging concentrations and deposition fluxes in the time period from 2012 to 2017 for each of the 12 sites, respectively.

The seasonal Mann–Kendall test (MK) (Helsel et al., 2006) was applied to the trend components time series extracted by STL decomposition from the original time series. Marchetto et al. (2013) showed that the non-parametric seasonal Mann–Kendall trend test is most powerful to detect monotonic trends, although differences between trend methods become negligible for long time series. The Sen’s method (Sen, 1968) was used to calculate the trend slope. Trends were considered significant if *p*-value < 0.05.

PCA is a method to reduce the number of dimensions and complexity in a data matrix and therefore to identify groups, patterns, and relationships between the variables (Le et al., 2017). In PCA, a data set containing correlated variables will be transformed into a new data set containing new orthogonal, uncorrelated variables called principal components (PCs) (Olsen et al., 2012). PCA was applied to the spatial data matrix as well as the data matrices of the seasonal and trend components time series. Precipitation and wind velocity were included into the PCA, since they are dominant meteorological variables determining natural gradients in a maritime environment.

Redundancy analysis (RDA) was applied to evaluate the relative influence of time series of anthropogenic emissions and meteorological factors (explanatory variables) on the station mean concentration time series of the trend component (response variables). RDA is a method combining multiple linear regression and PCA, to extract the variance in a set of response variables that can be explained by a set of explanatory variables (Borcard et al., 2011; Legendre & Legendre, 1998). Selected parameters were separated into three groups: (a) SO_4_^2−^ and total N; (b) As, Cd, Cr, Pb, Cu, Ni, and Zn, and (c) Hg. A RDA was performed for each group. Time series for the explaining meteorological factors (cloud cover, temperature, precipitation, wind velocity, sunshine duration, and humidity) stem from the weather station St. Peter-Ording (DWD, 2018). Additionally, the North Atlantic Oscillation (NAO) index was included as further explanatory variable (CRU, 2019; Jones et al., 1997). The spatially averaged distribution of the wind velocities were received from DWD (2018). The emission time series for Germany and Europe were taken from EEA (2018). To reduce the number of regression variables, a PCA was applied to extract a single emission factor for Germany and Europe for the emissions of group (a) and group (b), respectively. To avoid inconsistencies, all the time series were aggregated to yearly means for the period 1997 to 2016. A forward selection algorithm was used to extract those explanatory variables that have highest explanatory power and show lowest collinearity (Blanchet et al., 2008; Borcard et al., 2011). The data sets were log transformed, centered, and *z*-scaled before passed to PCA and RDA. Monthly averaged ammonia emission data were extracted from Backes et al. (2016).The relative contribution of the two sets of explanatory variables (emissions and meteorological factors) to the total variance explained by the set of response variables was evaluated by variation partitioning (Borcard et al., 1992; Peres-Neto et al., 2006).

Multiple linear regression was applied to estimate seasonal variation of ammonia concentrations in precipitation. The formula of the multiple linear regression model is

7 $$y=\beta_0+\beta_1X_1+\dots+\beta_{\mathrm n}X_{\mathrm n}+\varepsilon$$

where *y* is the predicted value of the dependent variable, *β*_0_ is the *y* intercept, *β*_1 to n_ are the regression coefficients of the independent variables *X*_1 to n_, and *ε* is the model error (Legendre & Legendre, 1998).

Statistical computations were implemented with the statistical open source software R (R Core Team, 2013) packages STPLplus (Hafen, 2016) and vegan (Oksanen et al., 2019).

## Results and discussion

### Dimension reduction — groups, patterns, and relationships between the parameters

**Fig. 2:**
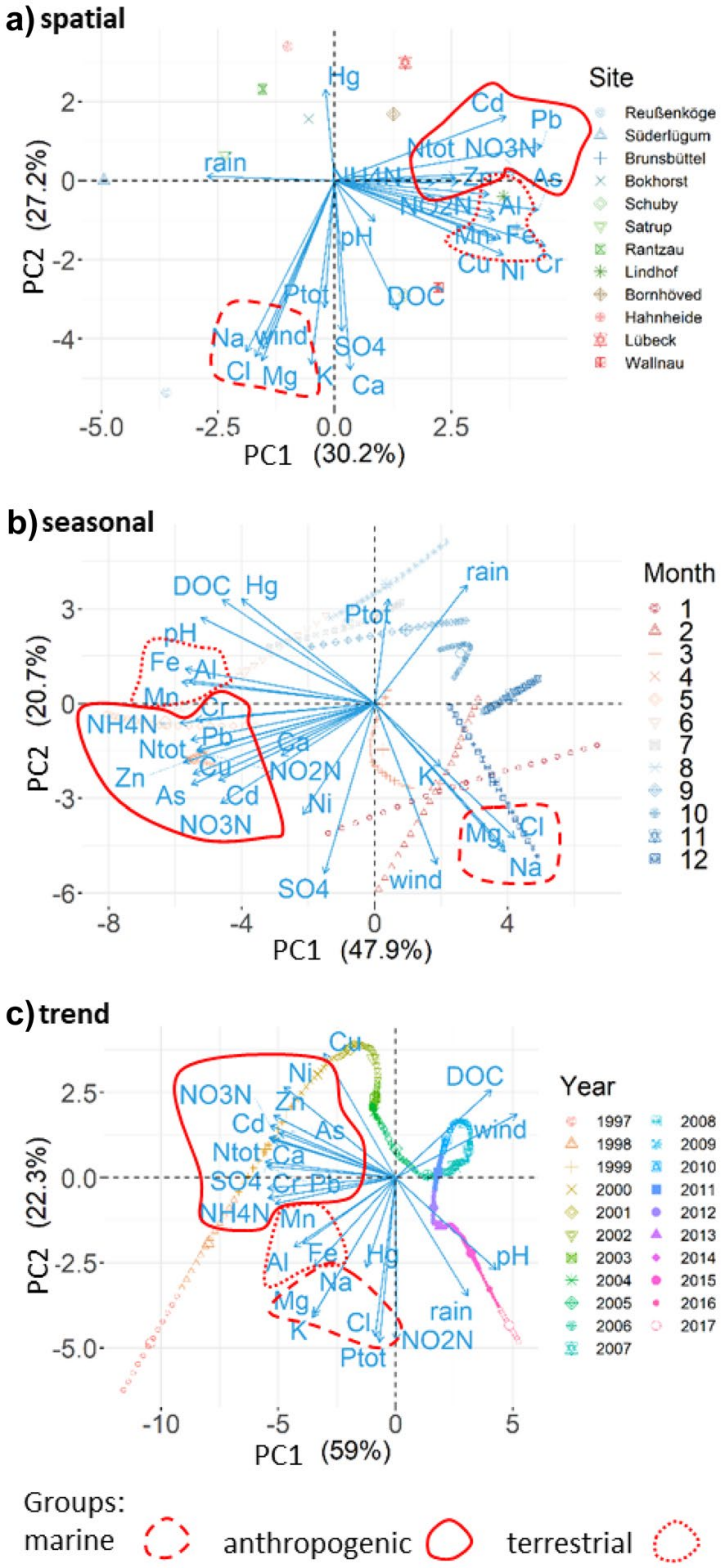
Principal component analysis of the **a** spatial data matrix as well as the time series of the **b** seasonal component and **c** trend component extracted from the original time series (1997 to 2017) by the STL method. Strongly related parameters are encircled and the identified groups are valid for the spatial, seasonal, and trend data matrix

Two main groups could be extracted from the parameters, based on ordination (PCA) (Fig. 2): (I) the marine group is formed by the elements Cl^−^, Na^+^, and Mg^2+^ and is related to natural processes. (II) The anthropogenic group is formed by the heavy metals Pb, Cd, As, and Zn and is related to altered patterns and processes due to anthropogenic emissions. As a subgroup, the terrestrial group is formed by the elements Fe, Al, and Mn, often referred as crustal elements. The assignment of Cu, Ni, and Cr to the anthropogenic or terrestrial group is less obvious. For the spatial PCA, they are related to the terrestrial group, but for the season and trend components, they are related to the anthropogenic group. The spatial, seasonal, or trend patterns of Hg are not related to any of the identified groups. The marine group is positively related to the wind velocity, which indicates the influence of sea spray. The anthropogenic group is inversely related to the precipitation. These three groups were valid for the spatial, seasonal, and trend data set, which underpins the role of fundamental processes for temporal patterns in depositions.

The anthropogenic group might be associated with anthropogenic emissions from traffic (mainly Pb: petrol and Zn: tires) and combustion for energy production and industrial processes (Huang et al., 2009; Schröder et al., 2016). As terrestrial, crustal elements, Fe, Al, and Mn, are often related to resuspension of soil-derived particles (Huang et al., 2009), but they can also originate from traffic as well as steel and production industry (Mijić et al., 2010). Cr and Ni are mainly related to combustion and industrial production and they are important constituents of many metal alloys (Huang et al., 2009; Mijić et al., 2010). Potential sources for Cu are traffic related, especially brake abrasion (Rajsic et al., 2008). But these elements can also be enriched in soils due to fertilizer application (Atafar et al., 2009; Mortvedt, 1995). This suggests that the elements of the terrestrial group are influenced primarily by anthropogenic emission sources, like traffic and combustion, and secondarily by resuspension of soil particles.

### Spatial distribution

#### Marine group

**Fig. 3:**
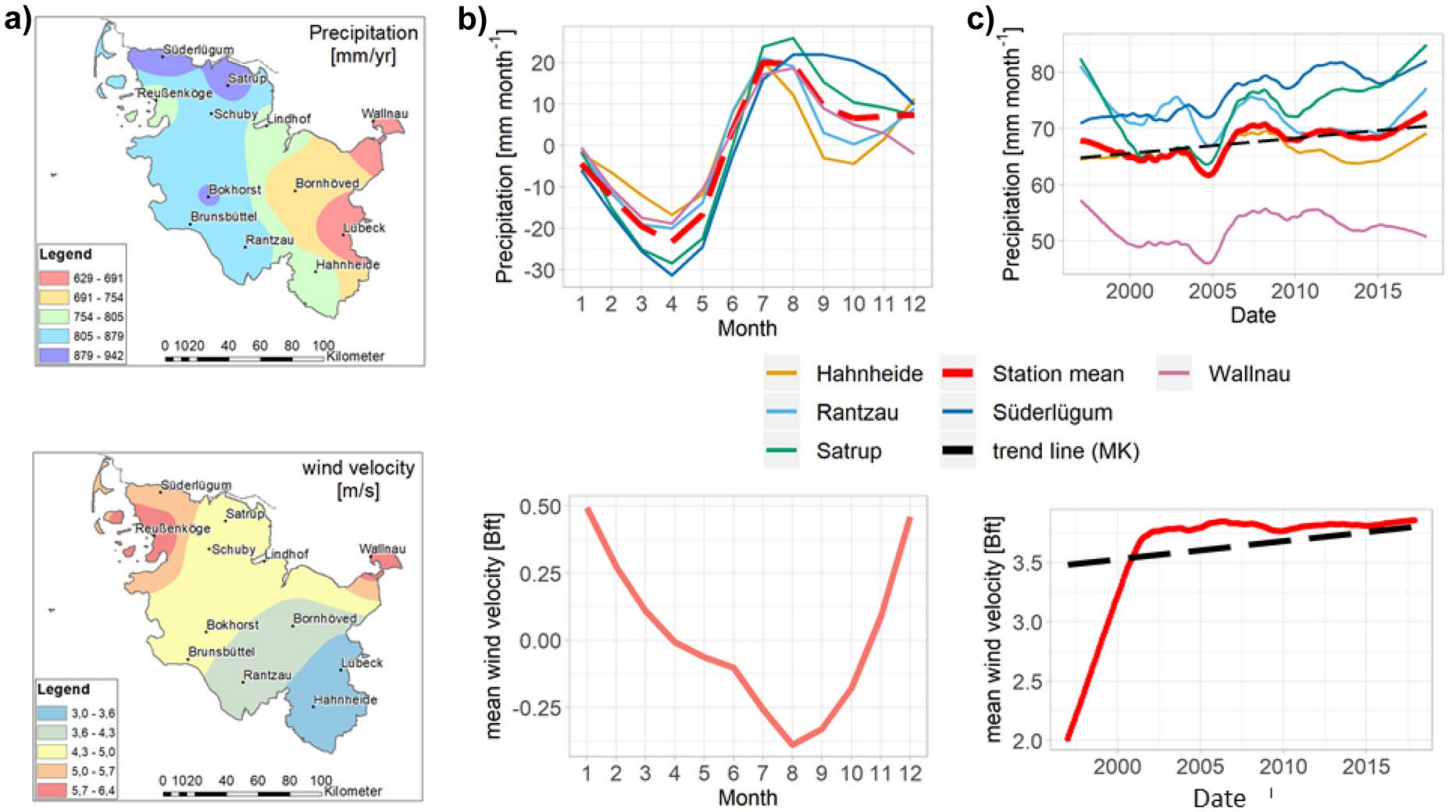
**a** Interpolated (inverse distance weighted; IDW) annual mean precipitation and wind velocity in the period 2012 to 2017. **b** Seasonal component and **c** trend component extracted from the precipitation and wind velocity time series (2012 to 2017) by the STL method

**Fig. 4:**
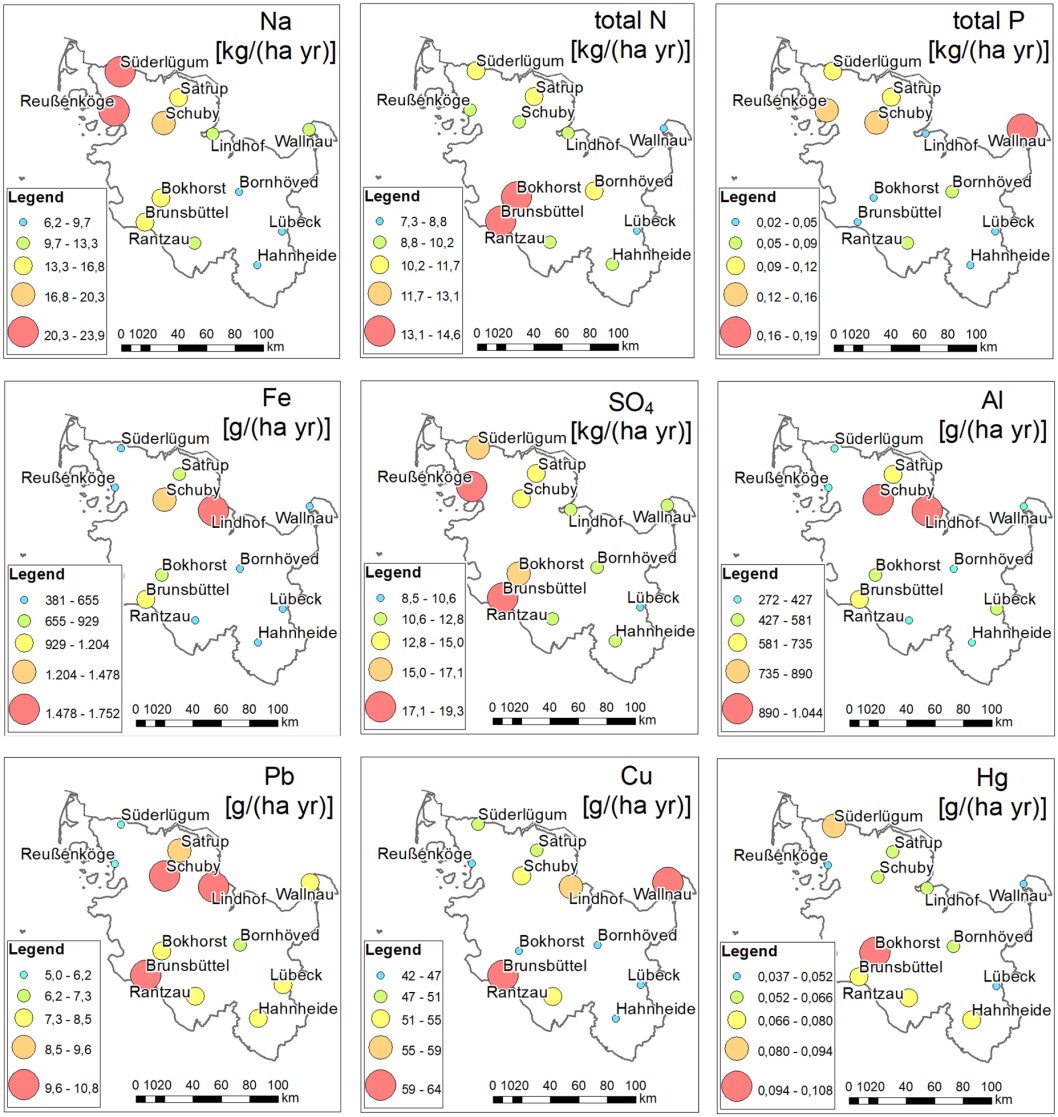
Annual mean deposition fluxes of selected parameters in Schleswig–Holstein, Germany, for the period 2012–2017. The selected parameters are representative for the groups identified by PCA (Fig. 2a)

The investigated region shows strong spatial gradients in precipitation and wind velocity (Fig. 3a). This can be attributed to the marine environment with prevailing westerlies (Ganea et al., 2019). The spatial patterns of the marine group are strongly related to the spatial wind velocity gradient (Fig. 3a), corresponding to the diminishing influence of sea spray with increasing distance to the coast line, especially of the North Sea (Neumann et al., 2016). Hence, the elements of the marine group show a strong spatial gradient with higher concentrations and deposition fluxes in the northwest and lower in the southeast (Fig. 4).

#### Anthropogenic and terrestrial group

The annual mean concentrations and deposition fluxes of the investigated metals averaged over all sites are listed in Table 3. The spatial patterns of the elements of the anthropogenic group (Fig. 4) are inversely related to the precipitation, which is highest in the west and lowest in the east (Figs. 2 and 3a). Therefore, lower metal concentrations and deposition fluxes were found at the northern sites, where the precipitation is high. While concentrations tend to be elevated for most of the heavy metals at the Baltic site Wallnau (Pb, Cr, Cd, Cu, Zn, As), deposition fluxes are not, reflecting the influence of the decreasing precipitation. The spatial patterns, related to these natural gradients, are altered by local emission sources. Most of the elements of the anthropogenic and terrestrial group show elevated concentrations and deposition fluxes around the site Lindhof in the east. On the one hand, this site is located in a high intensive agricultural area on sandy to loamy soils that are vulnerable to wind erosion (Duttmann et al., 2011). On the other hand, this site is located in the vicinity of the cities Kiel and Rendsburg as well as related traffic hot spots. Furthermore, the elements of the anthropogenic group show elevated depositions near the industrial site Brunsbüttel, where several refineries are located. Another potential emission source may be shipping emissions, due to combustion of shipping fuel (Viana et al., 2014). Highly frequented shipping routes are located close to the sites Lindhof, Brunsbüttel, and Wallnau (Neumann et al., 2018). Most of the heavy metals show only low to moderate spatial differences in the concentrations and deposition fluxes with a factor of approximately 1.5 to 2. Only the elements of the terrestrial group show stronger differences with a factor of 3. Therefore, under consideration of the dominating westerlies, background concentrations may be determined by long range distance transport of other industrial hot spots in Germany and Europe (UBA, 2018).

**Table 3:** Averaged concentrations and deposition flux of heavy metals and Al in the precipitation in the period 1997 to 2017. The parameters are ordered based on moles

	Concentration		Dep. flux
Parameter	µg L^−1^	µmol L^−1^	g ha^−1^ yr^−1^
Al	64.302	2.382	521.09
Fe	93.700	1.673	781.72
Mn	8.738	0.159	72.47
Zn	10.324	0.158	80.34
Cu	6.427	0.101	51.17
Ni	0.514	0.009	4.15
Pb	1.030	0.005	8.02
Cr	0.250	0.005	2.00
As	0.134	1.791 10^−3^	1.08
Cd	0.044	3.957 10^−4^	0.36
Hg	0.008	4.043 10^−5^	0.07

#### Sulfur, nitrogen, and phosphorus

The annual mean concentration of total N is 1.3 mg N L^−1^ and the deposition flux is 10.4 kg N ha^−1^ year^−1^ in the period 2012 to 2017. NH_4_^+^ is the dominant nitrogen species with 63%, followed by NO_3_^−^ with 36% and NO_2_^−^ with 1%. The nitrogen species are associated with the anthropogenic group, reflecting the impact of anthropogenic emissions on their concentrations and deposition fluxes (Fig. 2). The relatively evenly spatial distribution of nitrogen deposition can mainly be attributed to high background ammonia emissions from livestock farming, since agriculture is the dominant land use in the region of Schleswig–Holstein with approx. 70% (Jacobsen et al., 2019). Furthermore, EEA (2018) estimated that approx. 90% of the total NH_3_ emissions of Germany could be attributed to agricultural emissions. Nevertheless, similarly to the anthropogenic group, highest NH_4_^+^ and hence total N concentrations and deposition fluxes were found at the industrial site Brunsbüttel, highlighting the potential importance of local industrial emission sources also for nitrogen (Fig. 4). In addition, the shipping sector may contribute to elevated atmospheric depositions at coastal sites, since shipping emissions contribute to the nitrogen deposition with 13% in the North Sea and 16% in the Baltic Sea (Neumann et al., 2018) and for Sulfur with 6% and 8% (Jalkanen et al., 2016).

The spatial patterns of other elements, like Ca^2+^, K^+^, total P, and SO_4_^2−^, are grouped somewhere between the marine and anthropogenic group, indicating that natural patterns are altered by anthropogenic emissions (Fig. 2a). In addition, the high fraction of the non-sea salt concentration of these elements also indicates an alteration of the natural patterns by anthropogenic emissions (77% nssCa^2+^, 74% nssK^+^, and 73% SO_4_^2−^). The general spatial patterns of SO_4_^2−^ and Ca^2+^ are similar to the marine group, but they also show elevated concentrations and deposition fluxes at the industrial site Brunsbüttel (Fig. 4). Instead, K^+^ and also total P that are part of many fertilizers show elevated concentrations and deposition fluxes in the northeast and east probably due to intense agriculture in the surroundings. Additionally, the PCA shows that the spatial patterns of total P are moderately related to the marine group and wind. The average total P deposition flux normalized by precipitation over all sites with a non-agricultural surrounding (*n* = 5; forest, industry, and city) is 0.06 kg P ha^−1^ year^−1^. In comparison, the average total P deposition flux over all sites with an agricultural surrounding is 0.014 kg P ha^−1^ year^−1^ (*n* = 7). Therefore, it can be assumed that the spatial variation in the phosphorus deposition is effected by local conditions, like agricultural management and local wind patterns. On the other hand, it has been shown that phosphorus is enriched in sea spray aerosols (Graham et al., 1979) and that sea spray aerosols can be an important phosphorus source for coastal areas (Myriokefalitakis et al., 2016; Vignati et al., 2010).

#### pH and H^+^ deposition flux

In the period 2012 to 2017, the annual mean pH in precipitation is 5.9 and the averaged deposition flux of H^+^ is 20.2 eq ha^−1^. The results of the spatial PCA reveal that the pH has high loadings on PC-axis 3, together with SO_4_^2−^, NH_4_^+^, total N, and Ca^2+^, explaining 18.8% of the total spatial variance. In general, the pH in precipitation is higher in the west and lower in the east (Fig. 4). In correspondence with the emission hot spot of SO_4_^2−^ and NH_4_^+^, highest pH was observed at the industrial site Brunsbüttel. The ratio of nitrate to non-sea salt sulfate in precipitation is a useful indicator of the relative contribution of sulfuric acid (H_2_SO_4_) and nitric acid (HNO_3_) to the acidity of rain water (Itahashi et al., 2018). The ratio of nssSO_4_^2−^/(NO_3_^−^ + nssSO_4_^2−^) is 0.45 and therefore relatively balanced, with a slight dominance of NO_3_^−^ as main contributor to acidity (Wang et al., 2012). Nevertheless, because of the homogenous spatial distribution of NO_3_^−^, there is no significant correlation with the spatial distribution of the pH. The average ratio of NH_4_^+^/nssBC over all stations is 3.8. Higher concentrations of NH_4_^+^ than nssBC suggest that NH_4_^+^ is the main contributor to neutralize acidity in rainwater. At all sites, the ratio of NH_4_^+^/nssSO_4_^2−^ was between 1.8 and 2.8, indicating that ammonia can neutralize all the H_2_SO_4_, forming (NH_4_)_2_SO_4_ particles (Pascaud et al., 2016). The significant correlations of the spatial distribution of the annual mean pH with SO_4_^2−^ (Spearman correlation *r* = 0.62, *p* < 0.05) and NH_4_^+^ (Spearman correlation *r* = 0.72, *p* < 0.05) emphasize the dominant influence of these parameters on the spatial variation of pH in precipitation.

### Seasonal variation

#### Marine group

**Fig. 5:**
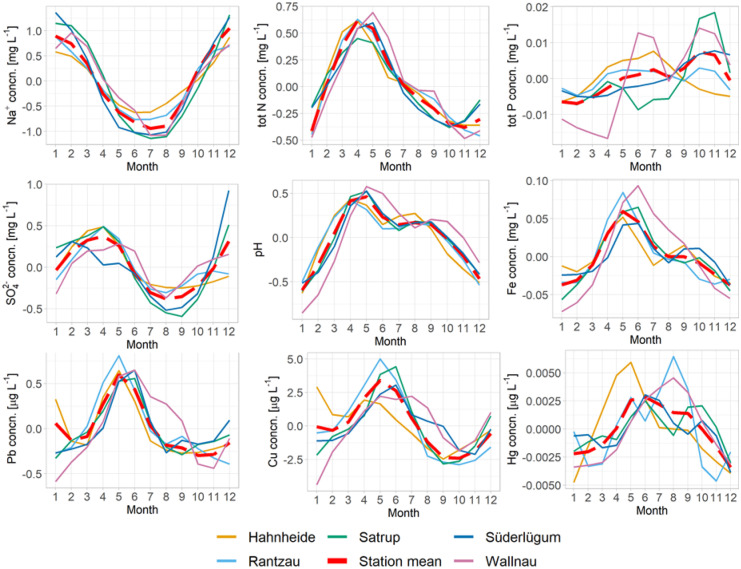
Monthly concentration time series of the seasonal component extracted by STL regression method for selected parameters (1997 to 2017). The selected parameters are representative for the groups identified by PCA (Fig. 2b)

The investigated parameters show different seasonal patterns. The seasonal patterns of the elements of the marine group (Na^+^, Mg^2+^, and Cl^−^) correspond very well with the mean wind velocity (Fig. 3b). Highest concentrations were found in the winter month and lowest during summer (Fig. 5). During the winter month, high wind velocities prevail and storm frequency are high (Dangendorf et al., 2014), resulting in a stronger influence of sea spray.

#### Anthropogenic group and nutrients

The seasonal variation of the concentrations of the elements of the anthropogenic group shows a moderate negative relationship to the precipitation, together with the nitrogen species (Fig. 2b). Although different emission sources could be expected, the seasonal patterns are very similar for all heavy metals and over all sites, except for Ni and Cd. Most of the heavy metals and the nitrogen species show a concentration maximum around April to May, corresponding very well with the precipitation minimum during this time (Fig. 5). While the concentration minima around October to November is shifted in comparison to the precipitation maximum in August, the deposition fluxes are shifted according to the seasonal variation in precipitation, with highest deposition fluxes around June to July and lowest around February to March. The spatial and seasonal behavior of Hg is not related to the other metals, but there is a weak to moderate relation to pH and DOC (Fig. 2a, b). The amplitude of the concentrations and deposition fluxes of the heavy metals and nitrogen species is in a narrow range of + / − 30 to 48% of the average. This range is in accordance with amplitude of the seasonal variation of the precipitation of + / − 30% (Fig. 2b).

**Fig. 6:**
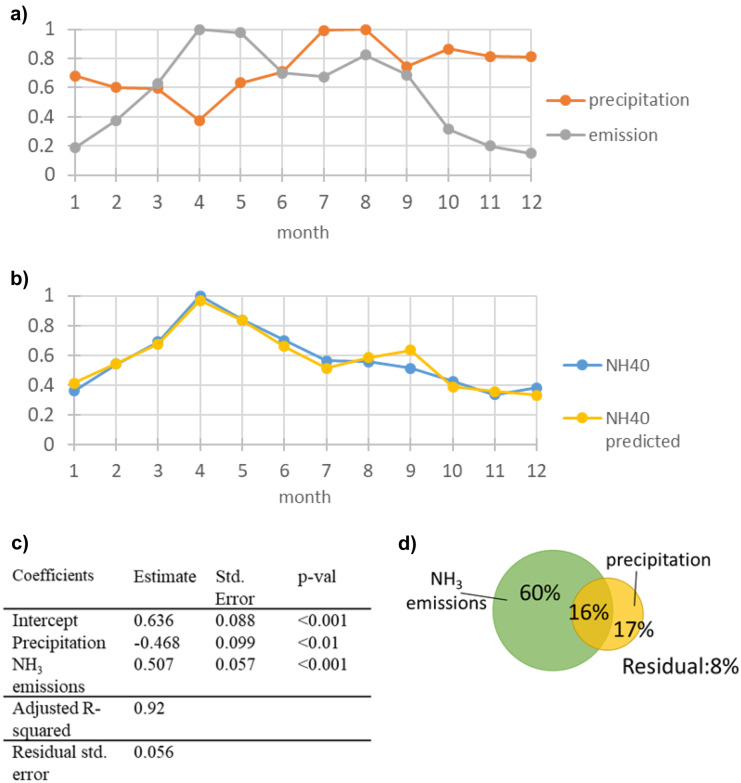
Results of the modelling of the seasonal component of the NH_4_^+^-N concentrations. **a** Standardized predictor variables: monthly averaged precipitation and NH_4_^+^-emission data. **b** Measured and predicted NH_4_^+^-N concentrations of the seasonal component (standardized). **c** Summary table of the regression models results. **d** Venn diagrams displaying the results of the variation partitioning analysis. The size of the diagrams is scaled to the total variance explained

The results of the PCA indicate that the precipitation is one important factor to describe the seasonal patterns of the anthropogenic group. Beside meteorological factors, seasonal patterns of the emissions may play an important role. According to the forward selection, monthly averaged precipitation and NH_3_ emission time series were used as explanatory variables in a multiple regression model to estimate ammonia concentration in precipitation (*p*-value < 0.001, Fig. 6c). High ammonia concentrations in April and May are favored by low precipitation and high agricultural NH_3_ emissions during this time, due to slurry application (Wagner et al., 2017) (Fig. 6a, b), while high NH_3_ emissions in August are compensated by high precipitation, resulting in falling ammonia concentrations until the minima in November to December is reached. According to the results of the variation partitioning, the seasonal variation of the ammonia concentrations is dominated by the seasonal patterns of the emission (60%), although the precipitation has a considerable part on the seasonality with 17% of the total variation explained.

The seasonal patterns of total P showed a relatively strong variation from site to site, emphasizing the potential importance of local effects like agricultural management and wind patterns on the total P concentrations and deposition fluxes.

#### pH and H^+^ deposition flux

The seasonal variation of the pH was also strongly correlated with the seasonal development of the NH_4_^+^/nssSO_4_^2−^ ratio (Pearson correlation *r* = 0.86, *p* < 0.05). The pH in precipitation reached its maximum of 6.5 together with the NH_4_^+^/nssSO_4_^2−^ ratio of 2.7 in April to May. In spring, NH_3_ emissions were at their maximum and the available amount of NH_4_^+^ to neutralize SO_4_^2−^ is twice as high as in winter, leading to highest pH values during the year. The minimum of the NH_4_^+^/nssSO_4_^2−^ ratio was reached in December with 1.4. During winter, the SO_2_ emissions were elevated, because of the heating period, and agricultural ammonia emissions were at their minimum, leading to lowest pH values of 5.4 during the year.

### Trends in depositions from 1997 to 2017

For the 24 investigated parameters, 21 and 20 significant trends for concentration and deposition flux could be determined, respectively (Table 4). Of those, 4 and 17 substances exhibited increasing or decreasing significant trends for concentration, respectively. In the case of the deposition flux, 4 parameters show increasing and 16 decreasing trends.

**Table 4:** Trends estimated by seasonal Mann–Kendall test from the station mean trend component time series (1997 to 2017). The standard deviation is derived from the trends of the individual sites

	Concentration		Deposition flux		
Parameter	Slope		Slope		
			[mm 21 yr^−1^]	[% 21 yr^−1^]	
Precipitation			68.1 ± 87.6	8.8 ± 10.8**	
	[21 yr^−1^]	[% 21 yr^−1^]			
pH	0.719 ± 0.234519	13.7 ± 4.7**			**
	[mg L^−1^ 21 yr^−1^]	[% 21 yr^−1^]	[kg ha^−1^ 21 yr^−1^]	[% 21 yr^−1^]	
H^+^	−0.009 ± 0.003	−80.2 ± 12.5**	−0.006 ± 0.002	−83.0 ± 14.3**	
Cl^−^	0.207 ± 0.509	7.4 ± 14.5**	0.252 ± 0.513	13.5 ± 20.4**	
Na^+^	−0.239 ± 0.414	−13.3 ± 16.9**	−0.104 ± 0.296	−8.7 ± 20.3**	
K^+^	0.020 ± 0.091	8.9 ± 29.3*	0.036 ± 0.038	26.1 ± 18.8**	
Ca^2+^	−0.191 ± 0.005	−46.8 ± 15.6**	−0.129 ± 0.081	−48.5 ± 15.3**	
Mg^2+^	0.005 ± 0.067	−2.5 ± 28.2	0.001 ± 0.038	0.8 ± 20.2*	
SO_4_^2−^	−1.375 ± 0.275	−49.1 ± 6.8**	−0.702 ± 0.200	−40.9 ± 5.3**	
NH^4+^-N	−0.296 ± 0.075	−29.6 ± 6.2**	−0.095 ± 0.031	−16.7 ± 3.2**	
NO_2_^−^-N	0.004 ± 0.002	57.3 ± 33.5**	0.004 ± 0.001	108.6 ± 39.0**	
NO_3_^−^-N	−0.192 ± 0.042	−30.3 ± 5.3**	−0.101 ± 0.026	−26.1 ± 2.1**	
Ntot	−0.513 ± 0.072	−31.0 ± 4.0**	−0.254 ± 0.051	−25.5 ± 2.8**	
Ptot	0.002 ± 0.012	17.6 ± 136	0.003 ± 0.008	44.4 ± 254	
Al	0.019 ± 0.012	−31.3 ± 16.0*	−0.001 ± 0.013	−4.6 ± 32.3	
Fe	−0.017 ± 0.025	−21.9 ± 31.8*	0.003 ± 0.021	8.0 ± 39.1	
Mn	−0.006 ± 0.003	−48.5 ± 20.0*	−0.002 ± 0.002	−33.1 ± 15.2**	
	[µg L^−1^ 21 yr^−1^]	[% 21 yr^−1^]	[g ha^−1^ 21 yr^−1^]	[% 21 yr^−1^]	
As	−0.059 ± 0.028	−31.7 ± 13.5**	−0.024 ± 0.010	−22.1 ± 7.2**	
Cd	−0.068 ± 0.021	−68.4 ± 17.5**	−0.033 ± 0.015	−58.4 ± 17.8**	
Cr	−0.162 ± 0.058	−44.0 ± 13.2**	−0.063 ± 0.025	−30.4 ± 11.2**	
Pb	−1.976 ± 0.330	−80.4 ± 11.6**	−0.949 ± 0.228	−69.2 ± 10.3**	
Cu	−6.421 ± 2.846	−51.9 ± 19.6**	−3.028 ± 1.517	−42.7 ± 13.8**	
Ni	−1.049 ± 0.269	−79.3 ± 6.5**	−0.672 ± 0.105	−80.4 ± 3.3**	
Zn	−7.236 ± 3.286	−47.8 ± 20.6**	−3.225 ± 2.336	−37.5 ± 20.0**	
Hg	−0.001 ± 0.002	−6.1 ± 23.8	0.000 ± 0.001	2.1 ± 33.0	
DOC	0.098 ± 0.151	7.5 ± 9.7**	0.136 ± 0.095	17.0 ± 11.2**	

Significant trend: **p < 0.05; *0.05 < *p* < 0.1

#### Marine group and precipitation

The northern sites (Süderlügum and Satrup) show a significant and notable increase in precipitation, ranging from + 16 to + 25% 21 year^−1^, whereas the southern and eastern sites show comparably small and contrary changes. The mean trend over all sites shows a significant increase of + 9% 21 year^−1^ (+ 68 mm 21 year^−1^) (Fig. 2). This is in accordance with Caloiero et al. (2018) who reported increases in precipitation for central and northern Europe (more than 20 mm/10 years) and seem to be connected to northern latitudes and exposure to the westerlies (BACC, 2008).

There is a notable increase of the wind velocities in the early part of the time series, followed by a period with comparably small changes. This pattern was also observed by Ganea et al. (2019), although they found slight decreasing trends for the longer time series of 35 years from 1983 to 2017. For the time period 1997 to 2017, the wind velocities show a significant increase of + 6.9% 21 year^−1^ (Fig. 2).

**Fig. 7:**
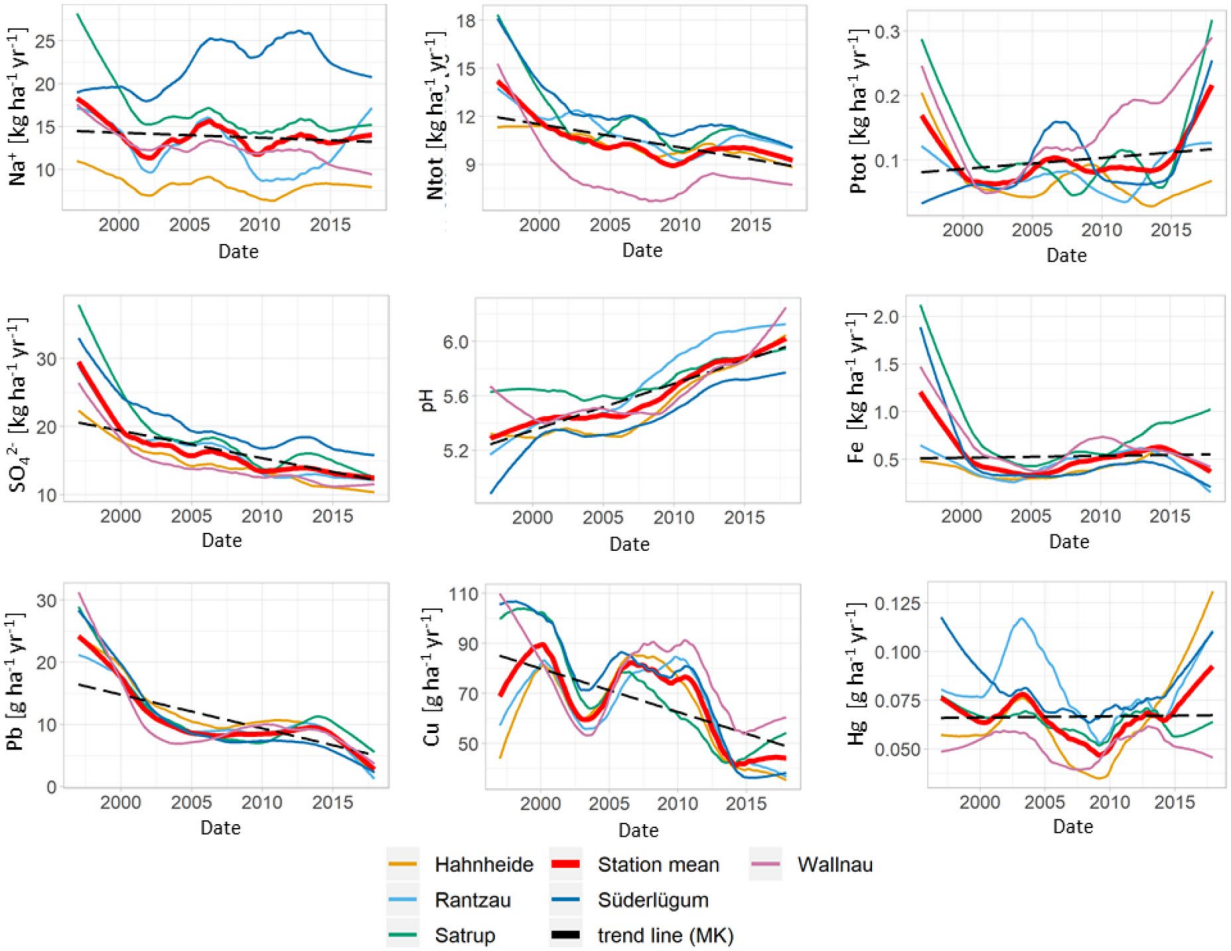
Monthly time series of the deposition flux trend component extracted by STL regression method for selected parameters (1997 to 2017). The selected parameters are representative for the groups identified by PCA (Fig. 2c). The black dotted line shows the Mann–Kendall trend line (MK)

The marine elements (Na^+^, Cl^−^, Mg^2+^) are grouped together by the PCA, also in the case of the trend component time series (Fig. 2c). Significantly decreasing trends of the concentrations and deposition fluxes were mostly observed at the sites closer to the Baltic Sea, while the site Süderlügum, which is located at the North Sea, shows increasing trends (Fig. 7). Although the precipitation trend is increasing at the sites Süderlügum and Satrup, they show contrary trends in the concentrations and deposition fluxes of the marine elements. This may be attributed to the diminishing influence of sea spray, whose influence dominates at areas within approximately 25 km from the coastline in the main wind direction (Chen et al., 2016).

#### Anthropogenic group and nutrients

**Fig. 8:**
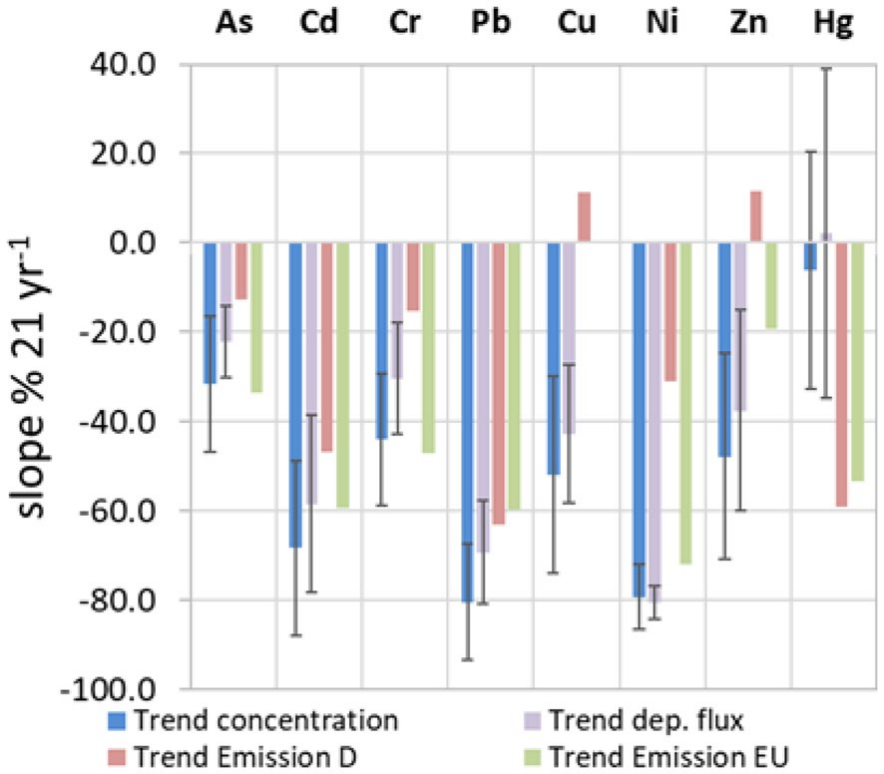
Comparison of the relative slopes (seasonal Mann–Kendall trend test) of the concentrations and deposition fluxes of the station mean trend component with the emission reductions reported for Germany (D) and the EU (EEA, 2018). Error bars indicate the standard deviation in respect to the individual sites

The elements of the anthropogenic group show considerable decreasing trends in concentrations and deposition fluxes in the period of 1997 to 2017 (Fig. 7 and Table 4). Pb shows the strongest decreasing relative trend slope for the concentration with 80% 21^−1^ year^−1^ and As the smallest with 30% 21^−1^ year^−1^. The terrestrial group shows weak decreasing trends and, except for Mn, no significant trends for the deposition flux. The relative trends of the heavy metals correspond with the reported European emission reductions (Fig. 8), implying that the heavy metals are strongly influenced by long range distance transport.

The trend component time series of total N, NH_4_^+^, and NO_3_^−^ and SO_4_^2−^ are closely grouped together with the anthropogenic group, indicating a strong influence of anthropogenic emissions on their temporal development (Beyn et al., 2014). Their concentrations and deposition fluxes are decreasing significantly at all sites and the magnitude of the decreasing relative trends corresponds well with the reported emission reductions for Germany (EEA, 2018). The decreasing trends of the nitrogen species of approximately -30% 21^–1^ year^−1^ for the concentrations and − 25% 21^–1^ year^−1^ for the deposition flux correspond well with the reduction of the summed NO_x_ + NH_3_ emissions of − 35% for Germany in the last two decades. The reduction of the emissions can mainly be attributed to NO_x_ reductions, while NH_3_ emissions were almost stable. Also, the decreasing trends of the concentrations and deposition flux of SO_4_^2−^ of -49% 21^–1^ year^−1^ and − 40% 21^–1^ year^−1^ correspond well with the relative SO_x_ emission reductions of Germany of − 53%, implying that the trends of SO_4_^2−^ and the nitrogen species are more influenced by local and regional emission sources. Conversely, the trend component time series of NO_2_^−^ is positively related to the precipitation and pH. All sites show significantly increasing trends, with approximately + 56% 21 year^−1^ for the concentrations and + 108% 21 year^−1^ for the deposition flux.

As it is the case for the spatial PCA, the trend component time series of total P is strongly related to the marine group. Although the overall trends in concentrations and deposition flux of total P are not significant, increasing trends have been found for total P concentrations and deposition fluxes at the sites close to the sea, especially at the Baltic site Fehmarn. On the one hand, these increasing trends may be attributed to the intensive agricultural surrounding of these sites and increasing mean wind velocities, favoring wind erosion and sea spray input. On the other hand, increasing P contents in the water column of the Baltic Sea have been reported for the same period of time, which may lead to increased P concentrations in sea spray (Savchuk, 2018; Stigebrandt et al., 2013).

#### pH and H^+^ deposition flux

Significant increasing trends of pH, ranging from + 6 to + 20% 21 year^−1^, respectively, decreasing H^+^ concentrations, ranging from − 56 to − 91% 21 year^−1^, and decreasing deposition fluxes of H^+^, ranging from − 56 to − 98% 21 year^−1^, were recognized at all sites. These findings are in agreement with increasing pH trends in other neighboring European countries (Pascaud et al., 2016; Tørseth et al., 2012; Vet et al., 2014). In the last two decades, the mean pH in precipitation increased by + 0.7 units 21 year^−1^. The NH_4_^+^/nssSO_4_^2−^ ratio increased from 1.4 to 2.3, in the period 1997 to 2017, and is strongly correlated with the trend component time series of the pH (Pearson coefficient = 0.98, *p* < 0.05). Therefore, the increasing trends in pH can mainly be related to the decreasing trends of SO_4_^2−^ and NH_4_^+^ and the resulting shift in the NH_4_^+^/nssSO_4_^2−^ ratio, emphasizing the dominant influence on the temporal, seasonal, and spatial variation of the pH. Therefore, emission reductions of NH_4_^+^ and SO_4_^2−^ have to be balanced to avoid a shift to lower pH.

#### Linking atmospheric deposition to meteorology and emissions

RDA and variation partitioning were applied to evaluate the relative influence of the temporal development of emission inventories and meteorological factors (e.g., like wind velocity, precipitation, or humidity) on the time series of selected response variables. To reduce the number of regression variables, a PCA was applied to extract a single emission factor for Germany and Europe (see “Statistical methods” section). The extracted emission factors explain 94% of the total variance of the emission data of group (a) and 92% of group (b), respectively. The results show that the influence of meteorological factors increases with increasing distance to the dominant emission sources.

**Fig. 9:**
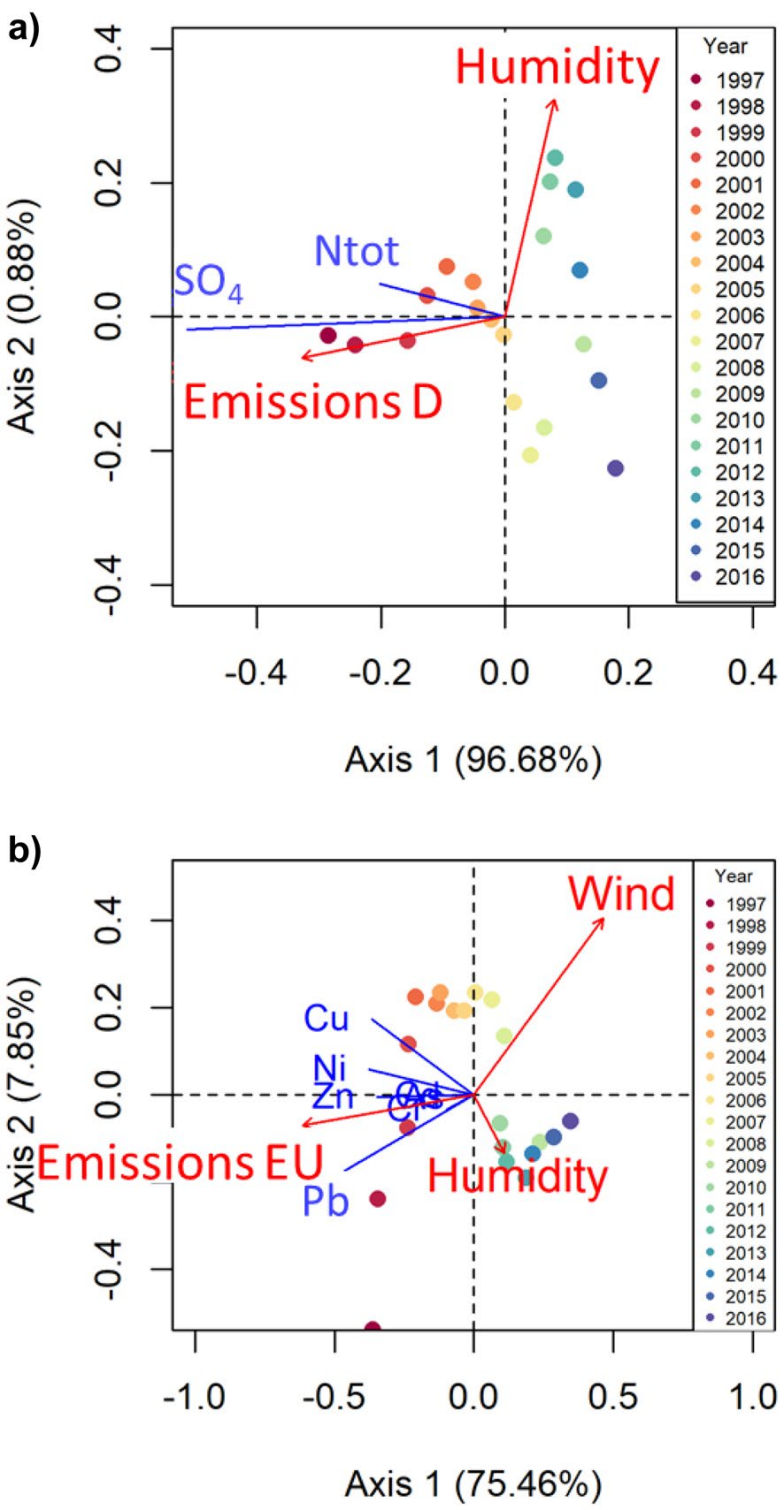
RDA triplot (scaling type 2) of the log transformed trend component time series of **a** SO_4_^2−^ and total N and **b** As, Cd, Cr, Pb, Ni, Cu, and Zn constrained by environmental variables (emission factor and meteorological factors). Blue lines are the response variables, red arrows are the environmental variables, and dots are the scores

**Fig. 10:**
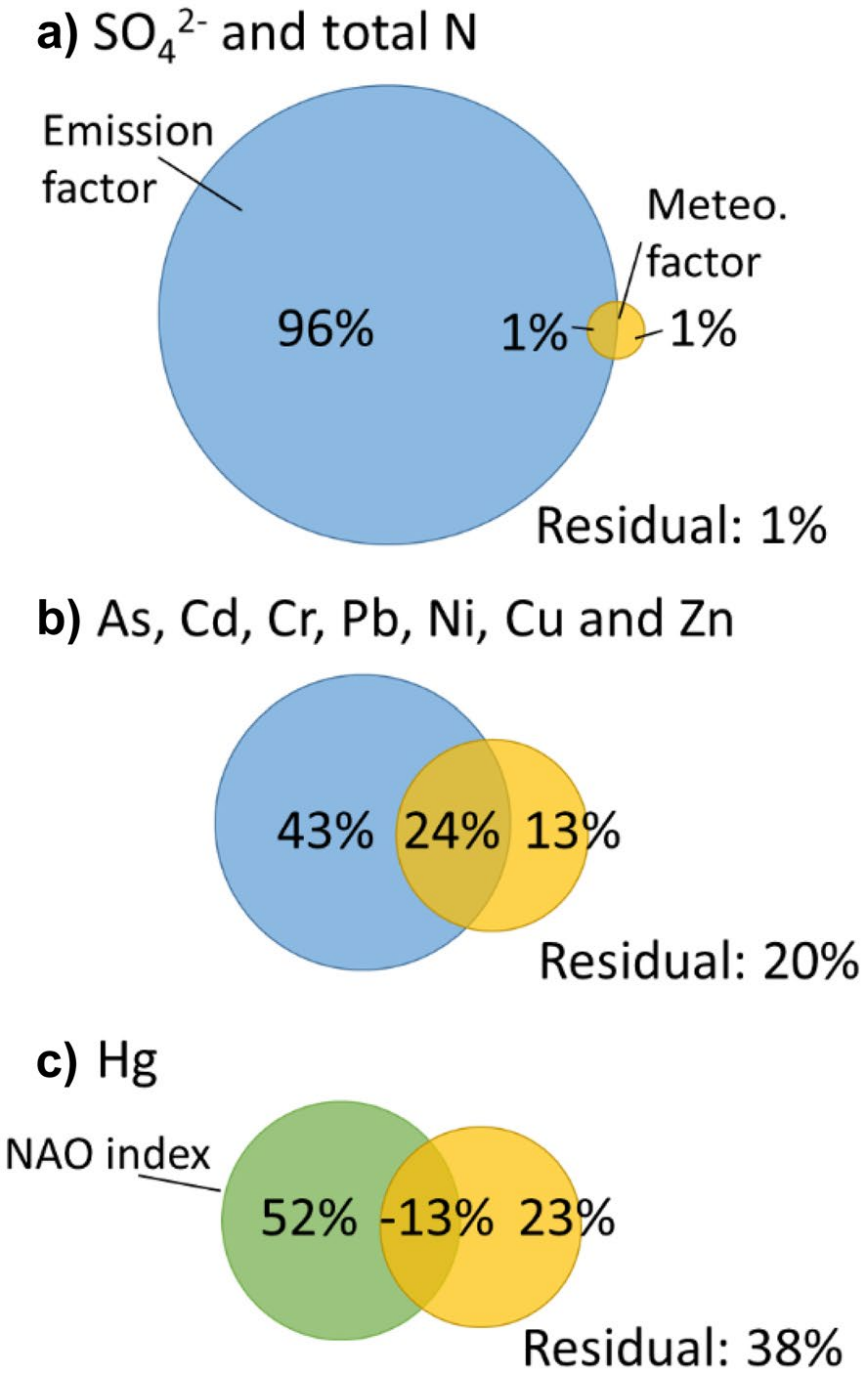
Venn diagrams displaying the results of the variation partitioning analysis. The size of the diagrams is scaled to the total variance explained

According to the forward selection, the emission factor of Germany and relative humidity were used as explanatory variables for the RDA model of the response variables SO_4_^2−^ and total N (see “Statistical methods” section). The emission factor and the response variables are strongly related to RDA Axis 1, while humidity is related to Axis 2. Axis 1 is most important and explains 97.8% of the variance of the response variables time series (Fig. 9a). The variation partitioning shows that 96% of the explained variance can be attributed to the emission factor of Germany (Fig. 10a). Therefore, the temporal development of the trend component time series of SO_4_^2−^ and total N is clearly dominated by the temporal development of local and regional emission sources.

In the case of As, Cd, Cr, Pb, Ni, Cu, and Zn, the emission factor of the EU, wind velocity, and relative humidity were detected as explanatory variables for the RDA. The RDA Axis 1 is most important, explaining 75.5% of the variance (Fig. 9b). The emission factor is strongly related to Axis 1, while mean wind velocity is related to both axes. The temporal development of the response variables corresponds positively with the emission factor and negatively with the wind velocity. Thus, Zn, Pb, and Ni contribute most to the Axis 1. Cu shows only a weak correlation with the emission factor and nearly no correlation with the meteorological factors. Cu has high loadings on the first unconstrained PC Axis 1 that explains 75% of the residual variance. Therefore, the temporal development of Cu cannot be explained by the available time series. Although there is a considerable amount of collinearity between the explanatory variables, the variation partitioning shows that 43% of the explained variance can be solely attributed to the emission factor, while 13% can be attributed to the meteorological factors mean wind velocity and relative humidity (Fig. 10b). Hence, the dominant factors for the temporal development of the concentration time series of As, Cd, Cr, Pb, Ni, and Zn are the emissions in Europe, but the meteorological factors wind and humidity have a considerable influence too, likely due to their impact on transport and dispersion (wind) and particle formation (humidity) (Amodio et al., 2014).

Hg does not show any correlation with the emission trends of Germany or the EU. Instead, the Hg trend component is correlated to the time series of the NAO index and wind velocity. Since only one response variable is considered, RDA is reduced to a multiple regression analysis. The resulting model explains 65.7% of the variance of the Hg time series. The temporal development of the Hg concentrations is positively related to the NAO index and negatively related to the mean wind velocity. The variation partitioning shows that 52% of the explained variance can be attributed to the temporal development of the NAO index and 23% to the mean wind velocity (Fig. 10c). The NAO index shows tendencies towards more positive NAO values, corresponding to stronger westerly winds (Hurrell et al., 2003). Hence, the positive correlation with the NAO index and the lack of significant correlations with the emission trends of Hg in the Europe and Germany implies that global emissions and transport of Hg are an important factor for the temporal development of the trend component of the Hg concentrations. This result is in good agreement with modelling results of Christoudias et al. (2012), who found that the NAO phase is significantly correlated with North American gas and aerosol tracer concentrations over the northwestern Atlantic Ocean and across northern Europe.

## Conclusion

Decreasing long-term trends in concentration and deposition flux could be identified for most of the identified parameters. Decreasing long-term trends of NH_4_^+^ and SO_4_^2−^ are strongly correlated with emission reductions in Germany, estimated by EEA (2018). The reduction of nitrogen emissions is related to reductions of industrial and transportation emissions, highlighting the need for the reduction of agricultural nitrogen emissions. The ratio of NH_4_^+^/non-sea salt SO_4_^2−^ determines the spatial distribution as well as seasonal and long-term development of the pH in precipitation.

Decreasing long-term trends of As, Cd, Cr, Pb, Ni, and Zn are dominated by emission reductions in Europe and meteorological factors. These findings support the importance of international conventions on emission reductions in order to achieve national environmental goals. Furthermore, it shows that meteorological factors can have a significant impact on the development of long-term trends. They can enhance or mitigate anthropogenic impacts on long-term trends.

The long-term trend of Hg shows no correlation to European and German emission inventories but to the North Atlantic oscillation (NAO). The annual NAO is a highly influential weather phenomenon as it controls the strength and direction of westerly winds in Europe. This correlation implies that both global emissions and transport of Hg are important factors for the temporal development of Hg depositions. For all waters in Germany, Hg concentrations in river biota exceed regulatory thresholds defined by the EU water framework directive. This highlights the importance for a global reduction of Hg emissions.

By the example of the seasonal variation of the ammonia concentrations, it has been shown that the knowledge of the seasonal variation of the emissions and driving meteorological factors is crucial to understand the seasonal variation of ammonia concentrations in precipitation.

The findings of this study are valuable for the evaluation of emission inventories and studies that are based on process-oriented emission and transport modelling. The time series decomposition into a seasonal and trend component enables to compare time series of selected measured parameters to available time series of emissions and meteorological factors. This technique aims to determine the decisive drivers and processes for regional depositions.

The identification of potential pollution hot spots and dominant drivers for the temporal development of seasonal variation and long-term trends of nutrients and heavy metals in atmospheric deposition helps local and regional decision-makers to develop and address expedient management strategies and measures. This approach will be extended in future studies to address the linkage of atmospheric deposition, emission factors, and meteorological factors to other environments, like concentration and loads in river basins.

## Data Availability

Data can be provided on request.
